# Ensuring quality of life in palliative care physiotherapy in developing countries

**DOI:** 10.3389/fresc.2024.1331885

**Published:** 2024-02-23

**Authors:** Babatunde Gbolahan Ogundunmade, Davidson Okwudili John, Nnenna Nina Chigbo

**Affiliations:** ^1^ Department of Physiotherapy, Jos University Teaching Hospital, Jos, Nigeria; ^2^ Department of Physiotherapy, Faculty of Health Sciences and Technology, David Umahi Federal University of Health Science, Uburu, Nigeria; ^3^ Department of Physiotherapy, University of Nigeria Teaching Hospital, Enugu, Nigeria

**Keywords:** palliative care, developing countries, quality of life, physiotherapy, service delivery

## Abstract

Palliative care (PC) focuses on the body, mind, and spirit and can also provide pain and symptom relief, clarifying and focusing the provision of care on the patient's desires and goals, and helping them understand their disease and its treatment plans. Although PC is widely recognized for its applicability near the end of life or during terminal illness, it is also applicable and beneficial for patients with diseases in their earlier stages. Near the end of life, palliative care often focuses on providing continual symptom management and supportive care. Although palliative care has been noted to improve some life expectancy, its primary aim is to improve quality of life via focusing on the comfort of the patient, maintaining dignity, reducing intensive care utilization, and avoidance of expensive hospitalizations. One major challenge to quality of life for patients in PC is the physical and functional decline that occurs with disease progression. These issues can be addressed by specialized PC physiotherapy. Uniform provision of high-quality PC services (and physiotherapy in palliative care) faces substantial challenges in resource-challenged settings, including low- and middle-income countries. When properly integrated into PC teams and adequately supported, physiotherapy within PC can address common symptoms (pain, breathlessness, weakness) and assist patients to remain in an adapted home setting to optimize their quality of life, safety, and dignity.

## Introduction

The role of palliative care (PC) is rapidly growing among patients with life-threatening illnesses to alleviate their symptom burden and improve quality of life (QoL). Palliative care is a multidisciplinary, holistic healthcare approach that helps patients and their families who are dealing with issues related to life-threatening or life-limiting illnesses be able to live better lives (1). This care approach aims to reduce suffering through the early detection, accurate diagnosis, and proactive treatment of pain and other issues, whether they be psychological, spiritual, or physical (1). The global challenges of an ageing population and chronic diseases are increasing the demand for high-quality palliative care.

The quality of life of patients and their families facing life-threatening and chronic illnesses should be a primary consideration during the management of their advancing medical conditions. Although palliative care services are often considered to follow the approach of hospice or end-of-life care, PC includes patients who do not necessarily have a terminal illness and/or a life-threatening illness, as well as those who continue to pursue curative treatment (2–5). End of life is defined as the period when a person’s advanced illness increases the likely result of death within the short term, leaving them without possibility of recovery (6). Although there is rarely a formal rule for eligibility for palliative care, an anticipated remaining life expectancy of 4–6 months or less is a common timeframe to engage with palliative care practitioners (7). In addition, palliative care practitioners focus on clarifying and aiming care towards the patient's life wishes and care goals while also helping them to understand treatment plans within the context of the reality of their medical situation.

A team-based approach is used in palliative care to help both patients and caregivers. It provides a network of support and leverages the various unique skills of the team members to enable patients to lead as full lives as possible until their death. Palliative care encompasses a variety of services provided to patients and their families by a range of experts, all of whom have equally vital roles and responsibilities. These professions include physiotherapists, doctors, nurses, support workers, paramedics, pharmacists, and volunteers. As palliative care is most effective in a team-based approach, Belgacem et al. (8) proposed that palliative care can be envisioned as a triangular relationship of equal participation between patients, informal caregivers, and healthcare providers to enhance the QoL of patients and caregivers as well as minimizing caregiver burden.

A critical barrier to coordinating and providing end-of-life care is inadequate communication; early discussion of prognosis and end-of-life care options helps facilitate earlier entry into hospice and palliative programs (9). Although challenging, one of the important tasks of the palliative care team is to provide thorough education on the disease trajectory and clarifying the anticipated risks and benefits of treatments. A common misconception held by patients and families is that enrolment in palliative care services hastens a person's death; however, evidence demonstrates that this is not the case and in fact may increase the lifespan as well as improving the quality of remaining life (10, 11). These gains in QoL are associated with focusing on patient comfort, maintaining dignity, preserving functional capacity, reducing intensive care unit stays, and avoidance of expensive and uncomfortable hospitalizations.

The model of care used to deliver the palliative care services can affect the quality of the care received by the patient. A qualitative study by Sagha Zadeh et al. (12) identified six key domains that organizations and practitioners should focus on to optimize quality of life in palliative care: (1) an organization's philosophy and mission; (2) organizational policies; (3) caregivers’ behaviours and practices; (4) symptom management; (5) facility design, operation, and management; and (6) patient, family member, and caregiver experience. Although comprehensive, direct contact (e.g., providing the service directly vs. directing another healthcare provider to deliver the services) PC services provided 9 months before death demonstrated improved caregiver satisfaction and odds of having a home death (as opposed to dying in a nursing facility), earlier PC services (initiated up to 24 months before death) also improved patient QoL, symptom management, and patient satisfaction (6). A key component of comprehensive PC services is education on the role and delivery of end-of-life care; when healthcare providers, patients, and informal caregivers are educated on PC, there is an improvement in patient symptom control and caregiver QoL (13).

High-quality PC is often complex and resource-intensive and the interdisciplinary care, compassion, and empathy required may be challenging to consistently and uniformly provide in health systems, organizations, or nations with limited resources. This may be especially impactful in nations with developing economies, where access to and delivery of high-quality PC may be restricted by limited funding, availability of qualified providers, and restrictive regulatory policies. These barriers may be further exacerbated for services that may not be commonly considered to be directly associated with disease management, such as physiotherapy for physical disability and progressive limitations to activities of daily living (ADLs) with chronic or advancing diseases. Uncontrolled symptoms and progressive disability are typical in all life-limiting conditions, especially in the months preceding death (14). Functional decline is one of the most distressing symptoms that patients with life-threatening or chronic diseases will experience (15). The purpose of this article is to provide perspectives on barriers and methods to providing access to physiotherapy for patients receiving palliative care in nations with developing economies.

## The role of physiotherapy within palliative care

The physical aspects of progressive illness frequently manifest as physical discomfort or pain; mobility and gait instability; coordination and balance challenges; muscle strength and flexibility deficits; limited physical activity tolerance; and easy fatigability. In light of these physical changes and subsequent impairments to ADLs in a previously active individual, physiotherapy can provide key support for patients in palliative care and their families (1). The holistic approach of physiotherapy can address common symptoms such as pain, fatigue, and dyspnoea, and thereby may improve functional capacity to retain/regain independence and dignity. Furthermore, palliative physiotherapy can assist patients in managing symptoms such as coughing and breathlessness. The restoration of movement and function may also assist in reducing the risk of injury/illness, promoting physical activity, and avoiding possible disease complications (16–18). However, in situations when function cannot be enhanced or restored, physiotherapy can help preserve function, teach adaptability, and help prevent potential adverse effects of illness or treatment.

## The value of palliative care physiotherapy

Patients enrolled in palliative care are assisted by physiotherapist interventions by learning self-care techniques and how to engage in meaningful activities through comprehensive evaluation and patient-centred treatment planning. Physiotherapists are skilled in functional assessment, communication, and movement analysis. They can help maintain or improve function when medical deterioration is inevitable and can help mitigate the shift towards functional decline (14). Aspects of physiotherapy such as symptom management, communication, care coordination, as well as counselling and education can be provided depending on the unique needs of each patient. Physiotherapy within PC can be utilized to provide functional rehabilitation, exercise prescription, and manual handling, as well as non-pharmacological pain and symptom management (15). Functional rehabilitation can be utilized to preserve or enhance physical independence, reduce dependence on caregivers, and promote physical activity and exercise by addressing gait, balance, transfers, and positioning. Non-pharmacological symptom management include interventions for pain, fatigue, lymphedema, and breathlessness. Exercise prescription includes individualized regimens designed to increase or preserve muscle mass and range of motion to translate into functional performance and controlling symptoms. Finally, assessment of manual handling aims at teaching caregivers and other medical professionals safe methods to assist with mobility tasks, ADL performance, and safe, comfortable positioning.

## Physiotherapy interventions within palliative care

The achievement of the best possible QoL for both the patient and their families is a primary focus of palliative care physiotherapy (19). Evidence demonstrates that palliative physical therapy can improve the function, independence, comfort, mood, and QoL of patients (20). In addition to the physical benefits, physiotherapists can help patients optimize and preserve their dignity despite fluctuations or deterioration in their condition; this is done through direct therapeutic interventions as well as providing communication, education, and collaboration among caregivers and the interdisciplinary team. Although most commonly delivered directly by physiotherapists, the physiotherapist may engage other team members or institutional and community-based partners as an extension of palliative care physiotherapy, which may be especially relevant in resource-challenged settings. There is increasing support for rehabilitation within palliative care; the World Health Organization (WHO) European Region has published a policy framework for integrating rehabilitation into palliative care services (21). This landmark document is a clear statement of the benefits and role of rehabilitation professionals within palliative care and could be utilized by low- and middle-income countries (LMICs) to facilitate/influence policy on integrating rehabilitation into their national palliative care program.

### Physical mobility, exercise, and fatigue management in chronic illnesses

Physiotherapists can provide their commonly accepted rehabilitative procedures including improving physical mobility (improving ambulation, aerobic capacity, and functional retraining), strengthening, range of motion, as well as balance and coordination. The process of functional retraining, endurance training, and participation in ADLs and physical activities may result in some temporary fatigue but is generally associated with improved QoL. Physiotherapists provide energy conservation and activity pacing guidance to facilitate patient participation in ADLs and compliance with prescribed disease treatment protocols. The skilled and strategic support of patient participation into daily activities can improve mood and self-efficacy as well as providing cardiopulmonary, cognitive, and musculoskeletal benefits. Physiotherapists can provide supportive care and preventative interventions to reduce the negative effects of immobility such as range of motion, positioning, and airway clearance techniques, which can affect multiple body systems including the musculoskeletal and cardiovascular system as well as mood and cognition.

Aerobic exercises essentially help recondition and improve physical fitness and are also relevant in the management of lymphedema. There is a robust and growing body of evidence for the safety and efficacy of exercise on aerobic capacity and muscular strength during and after completion of cancer treatment (22). In addition, progressive resistive and aerobic exercises have been found useful in contribution to overall health outcomes in HIV/AIDS. Exercise has been found to address the unwanted weight gains and improved the cardiovascular health in people living with HIV/AIDS (23, 24). The management of fatigue, a common symptom reported by patients in palliative care, is a point of focus by physiotherapists and is frequently treated by exercise and physical activity (25). Physical activity/exercise has demonstrated positive influence on functional independence, pain, and overall QoL (26). Muscle relaxation techniques can be initiated to enhance motor performance but may also improve sleep hygiene, both of which can improve fatigue.

### Respiratory physiotherapy

As dyspnoea and shortness of breath are common symptoms of end-stage disease [e.g., lung cancer, cardiovascular disease, chronic obstructive pulmonary disorder (COPD)], physiotherapist interventions for this in palliative care include but are not limited to chest physiotherapy, autogenic drainage, education on breathing techniques, and pulmonary rehabilitation, commonly known as respiratory physiotherapy (27). To clear the airways and drain the lungs, chest physiotherapy may use deep breathing, percussion, vibration, postural drainage, huffing, or coughing. Autogenic drainage is a very controlled technique of breathing that uses various breath-exhalation depths and rates to transport mucus up the airways, causing a cough that may be voluntary or involuntary (28). Although it may be used independently by the patient, it does require practice, focus, and effort. The patient can also be taught Active Cycle of Breathing Techniques (ACBT), an active breathing method, to increase ventilation, mobilize secretions in the lungs, and eliminate excess pulmonary secretions. It consists of breathing control, deep breathing exercise (thoracic expansion exercises), and huffing or forced expiratory techniques. When taught properly, these techniques for respiratory physiotherapy are simple to learn, do not require any special equipment, and allow the patient to practice on their own without supervision. Because of this, the methods are inexpensive, particularly in areas with limited resources such as LMICs. These respiratory physiotherapy techniques can improve airway clearance, reduce breathlessness, and preserve and improve a variety of lung functions to optimize pulmonary gas exchange for optimal cellular function, including neurologic and muscular activities, all of which can improve health outcomes (29, 30). Pulmonary rehabilitation has been shown to be effective in improving lung function. Even in cases of chronic respiratory failure, such as advanced COPD, improvements have been reported (31). Pulmonary rehabilitation is considered to be standard of care for those with at least moderately severe COPD (32). The goal of pulmonary rehabilitation is for the patient to become more physically active, maintain independence longer, improve physical endurance, reduce dyspnoea, and improve patient involvement in disease treatment adherence (33). Patients with COPD who were undergoing pulmonary rehabilitation did not show any significant worsening in exercise tolerance, dyspnoea, and health-related quality of life (HRQOL) over a period of 7 years (34).

### Symptom and pain management

An important component of physiotherapy is reducing pain through direct interventions, coping strategies, and pain education. Biophysical agents (colloquially known as modalities) such as transcutaneous electrical nerve stimulation (TENS), cryotherapy, and superficial and deep heat treatments may be beneficial to reduce or control pain. As there is some concern related to electrical and thermal agents’ effects on diseases (i.e., spread of cancer from vasodilation), collaboration with the interdisciplinary team prior to application of these agents is imperative. Physiotherapists can provide education on avoiding symptoms triggers including proper positioning for comfort and ease with engagement of ADLs. In addition, this can increase restful sleep, which can result in improved mood and cognition and reduce the need for sedatives or opiate pain medications. Finally, stretching and joint mobilization techniques can ease joint immobility, prevent/relieve contractures, and improve the patient's ability to use extremities for ADLs and participation in recreational activities.

As lymphedema can be a debilitating and distressing symptom that may develop at any stage of the disease progression or at any point in the continuum of care, proactive expert management is warranted to reduce discomfort, allow for optimal participation in ADLs, and improve overall QoL. Proper assessment and management can commence once venous thrombosis and extensive nodal disease are ruled out or properly managed. Periodic screening, self-monitoring, and patient education are crucial for achieving early identification and improved management of lymphedema and should be included in the physiotherapist's long-term plan of care. Complete decongestive therapy (CDT) is standard of care and includes manual lymphatic drainage; compression bandaging or application of multi-level lymphedema systematic pressure bandaging; exercise; as well as skin hygiene and nail care.

## Palliative care physiotherapy in resource-challenged settings

### Resource-challenged settings

Palliative care services are beneficial for those who have life-threatening illnesses; however, providing PC services to this population is hampered by a number of issues in resource-challenged or -limited settings (35, 36). Non-communicable disease burden is rising sharply in LMICs. There is a large discrepancy between the availability and demand for PC in these resource-challenged nations (37). LMICs, according to the World Bank, are nations with gross national incomes between $1,136 and $4,465 (38). Approximately two-thirds of newly diagnosed cancer cases will occur in LMICs by 2035 (40). The healthcare systems of LMICs will be severely strained by this trend, as most of these nations have limited organizational and financial capacities to handle this increasing burden. In nations with limited resources, treating such diseases creates a significant demand on health services, driving up direct and indirect healthcare expenses.

### Palliative care and physiotherapy in resource-challenged settings

There are over 40 million individuals globally who require PC; 80% of these individuals reside in LMICs, and only 12% of them receive PC (41). The majority of individuals who receive PC reside in high-income nations (42). The barriers to providing PC in general are especially impactful in resource-challenged settings such as LMICs. These barriers include limited availability of pain medications (e.g., morphine and other opioids) and other palliative treatments, resource limitations to develop a dedicated hospital palliative program/facility, restrictive or antiquated guidelines and regulations for a palliative service, and lower prioritization and perceived value of palliative care services (36, 37, 43–45). The burden of chronic diseases differs when considering LMICs vs. high-income nations, with increased incidence of HIV/AIDS, ischaemic heart disease, COPD, and tuberculosis in LMICs (46). As physiotherapy is not fully or consistently integrated into PC services, these barriers also affect the practice of palliative care physiotherapy resulting in care that is not efficient, economical, accessible, and equitable.

Some of these challenges facing PC provision and physiotherapy care within PC are as follows:

#### Personal barriers

The knowledge, attitudes, beliefs, skills, culture of patients and families, the general public, and healthcare professionals (HCPs) are the main focus of personal barriers. The most common issue influencing the provision of PC for patients is the limited knowledge of HCPs, families, and the general public of the role and impact of PC and its advantages for patients and healthcare systems (43, 47). Another issue is the inappropriate differentiation between PC and hospice/end-of-life care (47, 48).

Physiotherapists in resource-challenged settings such as LMICs often have limited knowledge of PC and inadequate training in their role on the PC team (49–52). According to Morrow et al. (50), physiotherapists manage patients in need of PC based more on their beliefs (including self-developed morals and values) than on knowledge. This might be the result of insufficient undergraduate and graduate training in PC physiotherapy (50, 52). Ihegihu et al. (53) observed that clinical physiotherapy students had poor knowledge of palliative care. Therefore, incorporating palliative care knowledge into entry-level educational programs appropriately may improve and maximize each student's comfort and skill with providing care to patients in PC (50), thereby increasing the workforce available to deliver PC physiotherapy.

#### Organizational barriers

Organizational processes, organizational culture, and inadequate or flawed infrastructure are examples of organizational impediments. The availability of PC in LMICs is hampered by organizational problems (44, 54). A lack of physical infrastructure (e.g., buildings, furniture, beds, and chairs) was cited as one of the main barriers to the provision of PC (44, 54). The delivery of satisfactory physiotherapy in palliative care may be hindered by profession-specific inadequate infrastructure, including insufficient space for physiotherapy clinical spaces (e.g., gyms) and therapy equipment.

There is a significant infrastructure shortage in community settings/rural areas where primary healthcare centres are located. The country's geography or residing in rural or isolated areas may make it more difficult to access PC services (47, 49). This makes it challenging for patients in need of palliative care who live in rural communities to get physiotherapy treatments when they need them, especially considering the advancing functional limitations these patients develop. Furthermore, in many developing nations there are few or no physiotherapists at the primary healthcare level due to the shortage of qualified physiotherapists (55).

#### Healthcare system barriers

Healthcare system barriers include problems with education, workforce development, service delivery, and organization-to-organization access. The biggest obstacle to the availability of PC has reportedly been identified as a lack of qualified or undertrained PC providers (56, 57). One aspect of this is the scarcity of physiotherapists (55), particularly those with training in palliative care (50, 58). HCPs lack of professional training programs, and basic staff training, is one of the gaps in the system. Odetunde et al. (52) found that just 19.2% of HCPs had received PC training, primarily in the form of lectures and short courses. Eke et al. (58) also reported in their study that as low as 10% of physiotherapists had previous training on PC, while Morrow et al. (50) reported that only 21% of physiotherapists had training in PC. Without proper training of physiotherapists in PC, it may be difficult to galvanize enough staff in this area of practice. Adequate training would create more awareness and understanding while stimulating the interest of physiotherapists in PC. Proper training would also provide physiotherapists with the required skill sets in the necessary competency areas to practice PC physiotherapy. This increased knowledge may indirectly influence the physiotherapists to consider practicing in PC physiotherapy, thereby positively affecting the PC physiotherapy workforce.

#### Policy and funding barriers

The foremost barrier to providing PC is considered to be the lack or shortage of funding (54). Research has also revealed that the absence of a comprehensive national PC plan (43, 54), inadequate or restrictive legislation and policy, a fragmented or weak healthcare system (43, 44), and lack of government support all have a negative impact on provision of PC to patients with cancer. In LMICs, there is often limited or insufficient health insurance coverage, thereby requiring that the impoverished people in these resource-constrained nations pay for healthcare services out of pocket, which may not be feasible or realistic. In LMICs, the average coverage for health insurance is 31.1%, with significant differences observed between national income categories (59). In lower middle-income nations, the average coverage of health insurance is 27.3%, but in low-income countries, it is 7.9% (59). Without sufficient payment means or insurance coverage for physiotherapy, patients requiring PC physiotherapy may find it difficult to get the required services. As many patients in LMICs are already impoverished, the resulting out-of-pocket spending for healthcare drives them further down the poverty line or forces them to not receive these vital services.

## The future of palliative care physiotherapy

The role of PC physiotherapy in optimizing functional independence and improving quality of life in PC patients cannot be underestimated. Nonetheless, PC physiotherapy is currently underutilized in LMICs and other settings with limited resources. Efforts to improve uniform access to high-quality PC physiotherapy should prioritize developing strategies to overcoming the barriers and facilitating the preparation of physiotherapists and their integration into PC teams.

Palliative care physiotherapy should be incorporated into entry-level and postgraduate physiotherapy curricula to close the knowledge gap and address the personal barriers associated with providing this type of care. For the purpose of raising awareness and altering attitudes about PC physiotherapy, it is imperative that physiotherapists, the general public, and patients receive adequate and ongoing education. Adequate training for HCPs has been proposed as a method to improve the PC workforce and enhance the quality of care (54). Therefore, additional training for all physiotherapists in PC care could increase the workforce while improving the quality of service. Creating a dedicated/specialized career pathway for PC physiotherapists is crucial. This will attract and empower young physiotherapists to realize that PC physiotherapy is a promising, fulfilling, and secure career path.

For the organizational facilitators, planned improvements to the physical buildings of healthcare facilities can play an important role in the provision of physiotherapy services in palliative care settings. Consequently, it is suggested that policymakers work with regional, national, and global organizations to get funds for enhancing the delivery of healthcare, and these plans should include physiotherapy as indicated by the WHO European Region document *Policy Brief on Integrating Rehabilitation into Palliative Care Services* (21). LMICs should also develop a strategy for innovative health financing with a focus on their local peculiarities. Increased participation of physiotherapists in palliative care teams and settings will be made possible by including physiotherapy as a major stakeholder in national PC policy. Adopting a multidisciplinary approach to PC care, with the physiotherapist being a key member, will not only improve patient outcomes and QoL but decrease the burden on healthcare costs in the long term by reducing adverse events through early PT intervention in PC, patient and family education, and self-care. Palliative care physiotherapy services can be covered by health insurance, and this can be accomplished by advocacy, engaging stakeholders, and negotiating for coverage. Improving and expanding PC physiotherapy research may be a useful tool for facilitating policy.

Finally, due to difficulties with transportation or low incomes, the majority of PC services in resource-challenged settings/LMICs are received in the home. One way to increase access to PC for patients living in remote locations might be to include PC within primary healthcare services (60), including PC physiotherapy. This would make it easier for patients and their families who reside in rural locations to obtain comprehensive treatment without being burdened by excessive personal costs (61). This has the added advantage of reducing the risk of needing costly institutionalizations or hospitalizations due to inadequate care being received at home.

## Conclusion

This paper discussed the challenges and potential facilitators for PC physiotherapy services in resource-challenged settings or LMICs. Clearly identifying the challenges at every stage might facilitate the availability of PC physiotherapy in these jurisdictions and serve as a framework for creating a model for PC physiotherapy service delivery. However, as the chronic disease population is different in LMICs than that of high-income countries, adaptations may need to be made when adopting policies or clinical guidelines originating in high-income countries. Palliative care physiotherapy is becoming increasingly adopted for treating patients globally; however, it is still not widely used in resource-challenged settings or LMICs. Developing strategies to enhance and integrate PC physiotherapy into healthcare systems in resource-constrained/low-income countries is made feasible by this examination of barriers and potential facilitators.

## Call to action

We recommend the establishment of comprehensive PC centres (inclusive of physiotherapist services), especially at the community level; this will improve accessibility to service. Developing countries should invest in bridging the identified barriers by educating patients, informal caregivers, and healthcare providers on the core values of palliative care, its care philosophy and operations, as well as the promising fiscal and healthcare quality benefits. Physiotherapists will encounter patients with fluctuating or declining function, and therefore should make home and environmental modifications a necessary component of their care, including considerations for easy fatigability and energy conservation. Due to the high incidence of dyspnoea and breathlessness near the end of life, it is likely that pulmonary rehabilitation techniques are underutilized by PC physiotherapists in the care of COPD. Physiotherapists play an important and unique role within the multidisciplinary palliative care teams; their treatment interventions can enhance quality of life in chronic conditions and at the end of life. Developing countries need to provide increased financial and logistical support for training and inclusion of physiotherapy within PC, including adequately equipping and training physiotherapists in key areas of palliative care practice, and facilitate physiotherapists’ contributions to improving the quality of life for all patients facing life-threatening or advanced chronic illnesses.

## References

[1] World Health Organization Palliative Care (2020). Available online at: https://www.who.int/news-room/fact-sheets/detail/palliative-care (assessed October 25, 2023).

[2] Gurfolino V Dumas L. Hospice nursing. The concept of palliative care. Nurs Clin North Am*.* (1994) 29(3):533–46. 10.1016/S0029-6465(22)02238-1 7522320

[3] Raffa CA (2003). Palliative care: the legal & regulatory requirements. Caring Nov. (2003) 22(11):6–9. 14658196

[4] Lubell J. Easing their pain. Palliative care grows despite reimbursement issues. Mod Health. (2010) 40(22):30–2. 20536102

[5] Batchelor NH. Palliative or hospice care? Understanding the similarities and differences. Rehabil Nurs*.* (2010) 35(2):60–4. 10.1002/j.2048-7940.2010.tb00032.x 20306613

[6] Health Quality Ontario. Team-based models for end-of-life care: an evidence-based analysis. Ont Health Technol Assess Ser. (2014) 14(20):1–49. 26356140 PMC4561363

[7] Jordan RI Allsop MJ ElMokhallalati Y Jackson CE Edwards HL Chapman EJ Duration of palliative care before death in international routine practice: a systematic review and meta-analysis. BMC Med. (2020) 18(1):368. 10.1186/s12916-020-01829-x 33239021 PMC7690105

[8] Belgacem B Auclair C Fedor MC Brugnon D Blanquet M Tournilhac O A caregiver educational program improves quality of life and burden for cancer patients and their caregivers: a randomised clinical trial. Eur J Oncol Nurs. (2013) 17(6):870–6. 10.1016/j.ejon.2013.04.006 23759361

[9] Eues SK. End-of-life care: improving quality of life at the end of life. Prof Case Manag. (2007) 12(6):339–44. 10.1097/01.PCAMA.0000300408.00325.1c 18030155

[10] Irwin KE Greer JA Khatib J Temel JS Pirl WF. Early palliative care and metastatic non-small cell lung cancer: potential mechanisms of prolonged survival. Chron Res Dis*.* (2013) 10(1):35–47. 10.1177/1479972312471549 23355404

[11] Temel JS Greer JA Muzikansky A Gallagher ER Admane S Jackson VA Early palliaitve care for patients with metastatic non-small cell lung cancer. New Engl J Med. (2010) 363(8):733–42. 10.1056/NEJMoa1000678 20818875

[12] Sagha Zadeh R Eshelman P Setla J Sadatsafavi H. Strategies to improve quality of life at the end of life: interdisciplinary team perspectives. Am J Hosp Palliat Care. (2018) 35(3):411–6. 10.1177/1049909117711997 28571497 PMC5866728

[13] Nevis I. Educational intervention in end-of-life care: an evidence-based analysis. Ont Health Technol Assess Ser. (2014) 14(17):1–30. 26339302 PMC4552297

[14] Cherny NI. “Physiotherapy in palliative care”. In: Cherny NI, editors. Oxford Textbook of Palliative Medicine, 6th ed. Oxford: Oxford Academic (2021). 10.1093/med/9780198821328.003.0024

[15] Nelson L Rankin J. Managing symptoms in palliative care. Frontline. (2019) (1). Available online at: https://www.csp.org.uk/frontline/article/managing-symptoms-palliative-care (accessed October 25, 2023).

[16] Byock IR Merriman MP. Measuring quality of life for patients with terminal illness: the Missoula-VITAS quality of life index. Palliat Med*.* (1998) 12(4):231–44. 10.1191/026921698670234618 9743822

[17] American Physical Therapy Association, APTA. What is physical therapy? A guide to physical therapist practise. Phys Ther. (2001) 81:21. 11175682

[18] Seamark D Ajithakumari K Devi PS Koshy R Seamark C. Palliative care in India. J R Soc Med. (2000) 93:292–5. 10.1177/014107680009300604 10911822 PMC1298030

[19] Wilson CM Stiller CH Doherty DJ Thompson KA Smith AB Turczynski KL. Physical therapists in integrated palliative care: a qualitative study. BMJ Support Palliat Care*.* (2022) 12(e1):e59–67. 10.1136/bmjspcare-2019-002161 32079576

[20] Putt K Faville KA Lewis D McAllister K Pietro M Radwan A. Role of physical therapy intervention in patients with life-threatening illnesses: a systematic review. Am J Hos Palliat Med. (2017) 34(2):186–96. 10.1177/1049909115623246 26722007

[21] World Health Organization Regional Office for Europe. Policy brief on integrating rehabilitation into palliative care services. Copenhagen: Licence: CC BY-NC-SA 3.0 IGO (2023).

[22] Allan J Buss LA Draper N Currie MJ. Exercise in people with cancer: a spotlight on energy regulation and cachexia. Front Physiol*.* (2022) 13:836804. 10.3389/fphys.2022.836804 35283780 PMC8914107

[23] O’Brien K Tynan AM Nixon S Glazier RH. Effect of progressive resistive exercise in adults living with HIV/AIDS: systematic review and meta-analysis of randomized trials. AIDS Care. (2008) 20:631–53. 10.1080/09540120701661708 18576165

[24] John DO Tella BA Olawale OA John JN Adeyemo TA Okezue OC. Effects of a 6-week aerobic exercise programme on the cardiovascular parameters, body composition, and quality of life of people living with human immune virus. J Exerc Rehabil. (2018) 14(5):891–8. 10.12965/jer.1836306.153 30443538 PMC6222150

[25] Mochamat Cuhls H Sellin J Conrad R Radbruch L Mücke M. Fatigue in advanced disease associated with palliative care: a systematic review of non-pharmacological treatments. Palliat Med. (2021) 35(4):697–709. 10.1177/02692163211000628 33765888

[26] Myrcik D Statowski W Trzepizur M Paladini A Corli O Varrassi G. Influence of physical activity on pain, depression and quality of life of patients in palliative care: a proof-of-concept study. J Clin Med. (2021) 10(5):1012. 10.3390/jcm10051012 33801357 PMC7958598

[27] Silva LES Cruz MDS Oliveira JD Ribeiro RGDS Lima PDO Quadros AAJ The function of the physiotherapist in palliative care and the resources used to improve the quality of life of oncological patients in terminal state. Res Society Dev*.* (2021) 10(16). 10.33448/rsd-v]01]6.23148

[28] Burnham P Stanford G Stewart R. Autogenic drainage for airway clearance in cystic fibrosis. Cochrane Database Syst Rev. (2021) 12(12):CD009595. 10.1002/14651858.CD009595.pub3 34910295 PMC8672941

[29] Lox CL Freehill AJ. Impact of pulmonary rehabilitation on self-efficacy, quality of life and exercise tolerance. Rehabil Psychol. (1999) 44:208–21. 10.1037/0090-5550.44.2.208

[30] Sachs S Weinberg RL. Pulmonary rehabilitation for dyspnea in the palliative-care setting. Curr Opin Support Palliat Care*.* (2009) 3(2):112–9. 10.1097/SPC.0b013e32832b7248 19381095

[31] Carone M Patessio A Ambrosino N Baiardi P Balbi B Balzano G Efficacy of pulmonary rehabilitation in chronic respiratory failure (CRF) due to chronic obstructive pulmonary disease (COPD): the Maugeri study. Res Med. (2007) 101(12):2447–53. 10.1016/j.rmed.2007.07.016 17728121

[32] Ries AL Bauldoff GS Carlin BW Casaburi R Emery CF Mahler DA Pulmonary rehabilitation: joint ACCP/AACVPR evidence-based clinical practice guidelines. Chest. (2007) 131(5 Suppl):4S–42S. 10.1378/chest.06-2418 17494825

[33] Carlin BW. Pulmonary rehabilitation: a focus on COPD in primary care. Postgrad Med*.* (2009) 121(6):140–7. 10.3810/pgm.2009.11.2082 19940424

[34] Foglio K Bianchi L Bruletti G Porta R Vitacca M Balbi B Seven-year time course of lung function, symptoms, health-related quality of life, and exercise tolerance in COPD patients undergoing pulmonary rehabilitation programs. Res Med*.* (2007) 101(9):1961–70. 10.1016/j.rmed.2007.04.007 17531455

[35] Geiling J Burkle FM Jr Amundson D Dominguez-Cherit G Gomersall CD Resource-poor settings: infrastructure and capacity building: care of the critically ill and injured during pandemics and disasters: CHEST consensus statement. Chest. (2014) 146(4 Suppl):e156S–67S. 10.1378/chest.14-0744 25144337 PMC6679686

[36] Poudel A Kc B Shrestha S Nissen L. Access to palliative care: discrepancy among low-income and high-income countries. J Glob Health. (2019) 9(2):020309. 10.7189/jogh.09.020309 31656599 PMC6812938

[37] Abu-Oda H Molassiotis A Liu J. Challenges on the provision of palliative care for patients with cancer in low- and middle-income countries: a systematic review of reviews. BMC Palliat Care. (2020) 19:55. 10.1186/s12904-020-00558-5 32321487 PMC7178566

[38] World Bank Group. World Bank country and lending groups: country classification (2017). Available online at: https://datahelpdesk.worldbank.org/knowledgebase/articles/906519-world-bank-country-and-lending-groups (accessed October 19, 2023).

[39] World Health Organization. Cancer. PRESS RELEASE NO: 263 (2018). Available online at: https://www.who.int/health-topics/cancer#tab=tab_1. (accessed November 11, 2023)

[40] Tefferi A Kantarjian H Rajkumar SV Baker LH Abkowitz JL Adamson JW In support of a patient-driven initiative and petition to lower the high price of cancer drugs. Mayo Clin Proc. (2015) 90(8):996–1000. 10.1016/j.mayocp.2015.06.001 26211600 PMC5365030

[41] Salikhanov I Connor SR Kunirova G Khashagulgova F Nazarova G Crape BL Challenges for developing palliative care services in resource- limited settings of Kazakhstan. Public Health Rev. (2023) 44:1605672. 10.3389/phrs.2023.1605672 37671066 PMC10476099

[42] Sallnow L Smith R Ahmedzai SH Bhadelia A Chamberlain C Cong Y Report of the lancet commission on the value of death: bringing death back into life. Lancet. (2022) 399:837–84. 10.1016/S0140-6736(21)02314-X 35114146 PMC8803389

[43] Fadhil I Lyons G Payne S. Barriers to, and opportunities for, palliative care development in the eastern Mediterranean region. Lancet Oncol. (2017) 18(3):e176–84. 10.1016/S1470-2045(17)30101-8 28271872

[44] Donkor A Luckett T Aranda S Phillips J. Barriers and facilitators to implementation of cancer treatment and palliative care strategies in low- and middle-income countries: systematic review. Int J Public Health. (2018) 63(9):1047–57. 10.1007/s00038-018-1142-2. 29974131

[45] Knaul FM Farmer PE Krakauer EL De Lima L Bhadelia A Jiang Kwete X Alleviating the access abyss in palliative care and pain relief—an imperative of universal health coverage: the lancet commission report. Lancet. (2018) 391(10128):1391–454. 10.1016/S0140-6736(17)32513-8 29032993

[46] Hajat C Stein E. The global burden of multiple chronic conditions: a narrative review. Prev Med Rep. (2018) 12:284–93. 10.1016/j.pmedr.2018.10.008 30406006 PMC6214883

[47] Rochmawati E Wiechula R Cameron K. Current status of palliative care services in Indonesia: a literature review. Int Nur Rev. (2016) 63(2):180–90. 10.1111/inr.12236. 26751254

[48] Bingley A Clark D. A comparative review of palliative care development in six countries represented by the Middle East cancer consortium (MECC). J Pain Symptom Manage. (2009) 37(3):287–96. 10.1016/j.jpainsymman.2008.02.014 18823750

[49] McDermott E Selman L Wright M Clark D. Hospice and palliative care development in India: a multimethod review of services and experiences. J Pain Symptom Manage. (2008) 35(6):583–93. 10.1016/j.jpainsymman.2007.07.012 18395401

[50] Morrow BM Barnard C Luhlaza Z Naidoo K Pitt S. Knowledge, attitudes, beliefs and experience of palliative care amongst South African physiotherapists. S Afr J Physiother. (2017) 73(1):384. 10.4102/sajp.v73i1.384 30135908 PMC6093105

[51] Alser AM Darwish AS Hussein KI Alabsi AA Alastal HM Sabbah LZ Knowledge, attitude and training physiotherapist palliative care—Gaza strip. Nutr Health Professions. (2020) 2(1). Available online at: https://dspace.alquds.edu/handle/20.500.12213/6308 (accessed November 12, 2023).

[52] Odetunde MO Owojuyigbe AM Aaron OI Odedeyi OT. Knowledge and attitude of health professionals on palliative care of terminally-ill patients in a tertiary health institution of southwestern Nigeria: a cross sectional study. Open J Med Res. (2022) 3(2):50–66. 10.52417/ojmr.v3i2.423

[53] Ihegihu EY Okoye CB Ihegihu CC Chukwuka BU Okoye EC Okonkwo UP Knowledge and attitude towards palliative care amongst clinical physiotherapy students in a Nigerian tertiary institution. Trop J Med Res. (2022) 21(2):11–6. 10.5281/zenodo.7295471

[54] Soto-Perez-de-Celis E Chavarri-Guerra Y Pastrana T Ruiz-Mendoza R Bukowski A Goss PE. End-of-life care in Latin America. J Glob Oncol. (2017) 3(3):261–70. 10.1200/JGO.2016.005579 28717769 PMC5493222

[55] Redda TH. The challenges of physiotherapy in a developing country such as Ethiopia—short communication. J Yoga Physiother. (2022) 9(4):555768. 10.19080/JYP.2022.09.555768

[56] Abdel-Razeq H Attiga F Mansour A. Cancer care in Jordan. Hematol Oncol Stem Cell Ther. (2015) 8:64–70. 10.1016/j.hemonc.2015.02.001 25732671

[57] Hannon B Zimmermann C Knaul FM Powell RA Mwangi-Powell FN Rodin G. Provision of palliative care in low- and middle-income countries: overcoming obstacles for effective treatment delivery. J Clin Oncol. (2016) 34(1):62–8. 10.1200/JCO.2015.62.1615 26578612

[58] Eke GK Ndukwu GU Chukwuma NS Diepiri BB. Knowledge and perception of health providers towards palliative care in Rivers State, Nigeria. Port Harcourt Med J. (2017) 11:156–60. 10.4103/phmj.phmj_26_16

[59] Hooley B Afriyie DO Fink G Tediosi F. Health insurance coverage in low-income and middle-income countries: progress made to date and related changes in private and public health expenditure. BMJ Glob Health. (2022) 7(5):e008722. 10.1136/bmjgh-2022-008722 35537761 PMC9092126

[60] Recoche K Lee S O'Connor M Ross-Heazlewood M Doherty V Hood K. Building palliative care capacity in rural health: a collaborative approach. Aust Nurs Midwifery J. (2014) 21(10):38. 24941569

[61] McCormick E Chai E Meier DE. Integrating palliative care into primary care. Mt Sinai J Med. (2012) 79(5):579–85. 10.1002/msj.21338 22976363

